# Changes in corneal curvature and astigmatism in senile cataract patients after phacoemulsification

**DOI:** 10.3389/fmed.2024.1481285

**Published:** 2024-10-31

**Authors:** Yan-Hui Xiao, Yue-Qi Liu, Zhi-Gang Chen

**Affiliations:** Department of Ophthalmology, The First Affiliated Hospital of Soochow University, Suzhou, China

**Keywords:** senile cataract, phacoemulsification, corneal curvature, axial length, astigmatism

## Abstract

**Purpose:**

Analysis of changes in corneal curvature and astigmatism after phacoemulsification for senile cataracts.

**Methods:**

Retrospective collection of clinical data from patients who underwent uncomplicated phacoemulsification at the First Affiliated Hospital of Soochow University. The changes in total corneal curvature, anterior surface curvature, posterior surface curvature, and astigmatism were measured by the Sirius system. The axial length was measured by Lenstar 900.

**Results:**

The total corneal curvature and anterior surface curvature at 3 months were all larger than those before phacoemulsification, and the difference was statistically significant (*p* < 0.05). Compared with preoperative results, there was no significant change in corneal posterior surface curvature and astigmatism 3 months after surgery (*p* > 0.05). Changes in corneal curvature and astigmatism were not significantly correlated with age at 3 months after surgery (*p* > 0.05). Postoperative astigmatism was increased with the growth of axial length, while corneal curvature was decreased (*p* < 0.05).

**Conclusion:**

Phacoemulsification can lead to increased postoperative corneal curvature in elderly cataract patients, and with the growth of the axial length, the corneal astigmatism was increased.

## 1 Introduction

With the continuous advancement of microsurgical techniques, artificial lenses, and intraocular lens (IOL) power measurement formulas, traditional cataract restoration surgery can no longer meet the living requirements of elderly patients, especially the presbyopia caused by monofocal IOL, which reduces the quality of life of patients. More and more elderly patients are choosing multifocal IOL. In order to meet their daily needs as much as possible, skilled surgical techniques, accurate measurement of eye parameters, and calculation of IOL power are required. At present, multifocal IOL is increasingly being used in clinical practice, improving the overall visual acuity of elderly cataract patients after surgical treatment and reducing glasses dependence after cataract surgery (1, 2). However, the presence of astigmatism affects the effectiveness of multifocal IOL. Astigmatism is a refractive error caused by the cornea and lens, which can lead to visual fatigue, ghosting, and decreased vision. After cataract surgery, the astigmatism caused by the cornea can often be greatly reduced due to the implantation of Toric IOL. However, if corneal astigmatism was ignored before surgery or appropriate treatment measures were not taken during surgery, it may affect the surgical outcome (3, 4).

At present, there are several methods for measuring preoperative corneal astigmatism: Pentacam anterior segment analysis system (Oculus Optikgeräte GmbH; Wetzlar, Germany), IOL-Master (Carl Zeiss Meditec AG, Jena, Germany), TMS-5 (Tomey Corporation, Nagoya, Japan), Sirius anterior segment analysis system (Oculus Optikgeräte GmbH; Wetzlar, Germany), and iTrace (Tracey Technologies Corp., Houston, TX) (5–8). At present, biometric instruments based on the Scheimpflug principle have been widely used in the field of ophthalmology. The Pentacam 3D anterior segment analysis system applies the Scheimpflug optical principle to obtain multiple images of the anterior segment through rotational tomography, obtain anterior and posterior surface curvature, total corneal thickness, ACD and other anterior segment parameters (9).

The Sirius system is a 3D anterior segment analysis system launched by Italian CSO company. It consists of a 360-degree rotating Scheimpflug camera and a Placido disc with 22 rings. It can capture 25 Scheimpflug images and a Placido image covering the anterior surface of the cornea within 1–2 s. The images include 35,632 and 30,000 data points, respectively. The anterior and posterior surface morphology, anterior chamber depth, and lens data of the cornea are calculated using proprietary software (10). Currently widely used in the diagnosis of anterior segment diseases, studies have shown that the Sirius system has high reproducibility and reproducibility in analyzing anterior segment parameters (11). Pentacam and Sirius system had good consistency in measuring anterior chamber depth and corneal curvature in cataract patients, providing a new option for measuring anterior segment parameters in clinical cataract patients (12, 13).

Previous studies have mainly observed the effect of Toric IOL on corneal astigmatism after cataract surgery, or the effect of cataract surgical incisions on astigmatism, but there has been relatively little research on postoperative corneal changes in elderly cataract patients. Research has found that the incidence of astigmatism is higher in elderly patients. After cataract surgery, corneal astigmatism shows a long-term inverse trend with age, but some scholars believe that there was no regular change (14, 15). Therefore, this study evaluated the changes in corneal biological parameters before and after surgery in age-related cataract patients using the Sirius system, exploring their characteristics and correlations, in order to provide assistance for clinical preoperative evaluation and IOL selection.

## 2 Materials and methods

### 2.1 Study design and patient selection

Patients who underwent cataract surgery at the First Affiliated Hospital of Suzhou University from July 31, 2017 to May 31, 2018 were collected through the hospital information system. Patients with II-IV grade nuclear hardness who were over 60 years old and had no surgical complications were included in the study. Patients with a history of ophthalmic surgery, corneal disease, uveitis, glaucoma, severe dry eye syndrome, posterior staphyloma, or poor fixation due to eye disease were excluded. The study adhered to the tenets of the Declaration of Helsinki. All patients have signed informed consent forms. After being reviewed and approved by the hospital ethics review committee.

### 2.2 Instrument and examinations

All patients underwent Lenstar LS900 and Sirius system examinations under natural pupil and relative darkroom conditions. The inspection was conducted by the same skilled inspector on the same day. Firstly, the patient underwent a Lenstar LS900 examination. After blinking several times, patients were instructed to place the jaw support on the lower jaw, pressed the forehead tightly against the forehead support, observed the instrument markings, and measured when the focusing aperture was at its minimum. Each measurement consists of 16 rapid and continuous scans with a total of three measurements taken and averaged. Then the corneal parameters were checked by the Sirius system. During the filming process, instructed the examinee to keep their eyes wide open, and avoid blinking. In order to ensure accurate and reliable inspection results, Sirius had strict quality control standards (included with the instrument): when the Scheimpflug image area was ≥90%, the center positioning was ≥90%, and the Placido disk coverage area was ≥80%, the inspection results could be accepted. Three measurements that meet the standards were taken. Three months after surgery, Sirius system examination was performed using the same operating method.

### 2.3 Surgical technique

After making a lateral incision at 2 o’clock, inject viscoelastic agent into the anterior chamber, and make a 2.2 mm single plane corneal incision at 10 o’clock. Continuous circular capsulorhexis with a diameter of about 6 mm was performed. The lens nucleus is emulsified and the cortex was removed using Alcon’s Centurion phacoemulsifier. Tecnis ZCB00 IOL (Abbott Medical Optics, Santa Ana, CA) were implanted in the lens capsule. All surgeries were performed by the same physician (Lu PR). Postoperative routine administration of tobramycin dexamethasone and levofloxacin eye drops were used for anti-inflammatory and anti-infective treatment.

### 2.4 Statistical analysis

SPSS version 19.0 (SPSS, IBM Corp., Armonk, NY, United States) was used for analysis. The normality of the continuous data was tested using Kolmogorov–Smirnov test. Normal distribution econometric data was represented by mean ± standard, Preoperative and postoperative corneal curvature and astigmatism changes were analyzed using paired *t*-tests. Spearman rank correlation is used to perform correlation analysis on various parameters. *p*-value of 0.05 is considered statistically significant.

## 3 Results

### 3.1 General results

A total of 76 cases (76 eyes) were collected in this study, including 46 males (46 eyes), 30 females (30 eyes), 38 left eyes, and 38 right eyes. Age ranges from 60 to 92 years old, with an average age of (71.2 ± 7.15) years. The mean axial length was 24.3 ± 1.98 mm (range from 21.17 to 29.09). The mean surgical duration was 7.03 ± 0.95 min, CDE value was 5.71 ± 5.35. There were no complications during the final follow-up after the surgery.

### 3.2 Changes and correlation of total corneal curvature, anterior surface curvature, and posterior surface curvature in cataract patients before and after surgery

As shown in Table 1, the median (range) flat axis of total corneal curvature before surgery was 43.42D (40.34 ~ 47.27D), the postoperative range was 43.47D (40.46 ~ 47.55D), the steep axis range was 44.24D (40.51 ~ 48.04D), the postoperative range was 44.29D (40.58 ~ 48.09D), the average range was 43.84D (40.43 ~ 47.58D), and the postoperative range was 44.83D (40.52 ~ 47.73D). Compared with preoperative, the total corneal curvature after surgery had increased, and the difference was statistically significant (*p* < 0.05). The preoperative range of corneal anterior surface curvature was 43.39D (40.37 ~ 47.42D), postoperative range was 43.53D (40.53 ~ 47.51D), steep axis range was 44.40D (40.55 ~ 48.18D), postoperative range was 44.49D (40.58 ~ 48.29D), average range was 43.88D (40.46 ~ 47.8D), and postoperative range was 43.92D (40.56 ~ 47.85D). Compared with preoperative, postoperative corneal anterior surface curvature also had increased, and the difference was statistically significant (*p* < 0.05). The flat axis range of corneal posterior surface curvature was −6.12D (−6.78--5.62D), postoperative was −6.12D (−7.08-5.23D), steep axis range was −6.47D (−8.8–5.92D), postoperative was −6.47D (−10.65–5.89D), average range was −6.31D (−7.52–5.84D), postoperative is-6.29D (−8.08-5.80D), and there was no statistically significant difference compared to preoperative (*p* > 0.05). The Spearman correlation analysis results showed a significant positive correlation between the anterior corneal surface curvature and the total corneal curvature, indicating that as the refractive power of the anterior corneal surface increases, the total corneal refractive power also increases; The posterior corneal surface refractive power was significantly negatively correlated with the total corneal refractive power, and significantly positively correlated with its absolute value, indicating that as the posterior corneal surface refractive power increases, the total corneal refractive power decreases, as shown in Table 2.

**Table 1:** Changes in corneal curvature in elderly cataract patients before and after surgery (mean ± SD).

Curvature		Preoperative	Postop 3 months	*t* value	*p* value
ACC	Flat value	43.65 ± 1.62	43.75 ± 1.65	−2.633	0.010*
	Steep value	44.50 ± 1.65	44.56 ± 1.68	−1.927	0.058
	Average value	44.06 ± 1.60	44.16 ± 1.64	−2.505	0.014*
PCC	Flat value	−6.16 ± 0.27	−6.13 ± 0.30	−1.469	0.146
	Steep value	−6.51 ± 0.45	−6.53 ± 0.57	0.281	0.780
	Average value	−6.34 ± 0.30	−6.33 ± 0.34	−0.141	0.888
TCC	Flat value	43.61 ± 1.59	43.71 ± 1.62	−2.721	0.008*
	Steep value	44.38 ± 1.63	44.49 ± 1.68	−2.884	0.005*
	Average value	44.00 ± 1.59	44.10 ± 1.63	−3.001	0.004*

Postop, postoperative; ACC, anterior corneal curvature; PCC, posterior cornea curvature; TCC, total corneal curvature; *paired *T*-test.

**Table 2:** Correlation analysis between corneal anterior and posterior surface curvature and total curvature.

Curvature	TCC			
	Preoperative		Postop 3 months	
	r_s_	*P* value	r_s_	*P* value
ACC	0.997	0.000	0.994	0.000
PCC (absolute value)	0.749	0.000	0.788	0.000

Postop, postoperative; ACC, anterior corneal curvature; PCC, posterior cornea curvature; TCC, total corneal curvature.

### 3.3 Changes and correlation of astigmatism in cataract patients before and after surgery

The preoperative median (range) of corneal surface astigmatism and total astigmatism were − 0.75D (−3.08–0.08D) and − 0.61D (−2.75–0.05D), while postoperative astigmatism were − 0.65D (−2.58–0.05D) and − 0.61D (−2.44–0.12D). Compared with preoperative astigmatism, the difference had not statistically significant (*p* > 0.05), as shown in Table 3. The Spearman correlation analysis results showed a significant positive correlation (*p* < 0.05) between total corneal astigmatism and anterior corneal surface astigmatism. The correlation with corneal posterior surface astigmatism was relatively small, indicating that corneal posterior surface astigmatism had a relatively small impact on total astigmatism, as shown in Table 4.

**Table 3:** Changes in corneal astigmatism in elderly cataract patients before and after surgery (mean ± SD).

Astigmatism (absolute value)	Preoperative	Postop 3 Months	*t* value	*P* value
AA (D)	0.83 ± 0.59	0.80 ± 0.56	0.795	0.429
PA (D)	0.35 ± 0.42	0.40 ± 0.61	−0.585	0.560
TCA (D)	0.78 ± 0.57	0.78 ± 0.50	0.132	0.974

Postop, postoperative; D, diopters; TCA, total corneal astigmatism; AA, anterior corneal astigmatism; PA, posterior corneal astigmatism.

**Table 4:** Correlation analysis between corneal anterior and posterior surface astigmatism and total astigmatism.

Astigmatism (D)	TCA			
	Preoperative		Postop 3 months	
	r_s_	*P* value	r_s_	*P* value
AA	0.848	0.000	0.796	0.000
PA	−0.026	0.822	0.053	0.650

Postop, postoperative; D, diopters; TCA, total corneal astigmatism; AA, anterior corneal astigmatism; PA, posterior corneal astigmatism.

### 3.4 Correlation between age, axial length, corneal refractive power, and astigmatism

The Spearman correlation analysis of age, axial length, corneal refractive power, and astigmatism were shown in Tables 5, 6. There was a mild positive correlation between preoperative age and total corneal curvature, but there was no significant correlation between the two after 3 months (*p* > 0.05). The axial length was negatively correlated with total corneal curvature, anterior surface curvature, and posterior surface curvature, indicating that as the axial length increases, corneal curvature decreased. The axial length was positively correlated with total corneal astigmatism and anterior surface astigmatism, indicating an increase in postoperative astigmatism as the axial length increased.

**Table 5:** Correlation between age and corneal curvature and astigmatism.

	Preoperative		Postop 3 Months	
	r_s_	*p* value	r_s_	*P* value
TCC	0.227	0.049	0.155	0.181
TCA	−0.018	0.874	0.015	0.895
ACC	0.225	0.050	0.143	0.219
AA	−0.120	0.303	−0.115	0.323
PCC	0.167	0.149	0.216	0.061
PA	0.079	0.497	−0.078	0.504

Postop, postoperative; D, diopters; TCA, total corneal astigmatism; AA, anterior corneal astigmatism; PA, posterior corneal astigmatism; ACC, anterior corneal curvature; PCC, posterior cornea curvature; TCC, total corneal curvature.

**Table 6:** Correlation between axial length and corneal curvature and astigmatism.

	Preoperative		Postop 3 months	
	r_s_	*P* value	r_s_	*P* value
TCC	−0.416	0.000	−0.406	0.000
TCA	0.251	0.029	0.283	0.013
ACC	−0.421	0.000	−0.420	0.000
AA	0.311	0.006	0.313	0.006
PCC	−0.367	0.001	−0.389	0.001
PA	−0.100	0.389	−0.051	0.663

Postop, postoperative; TCA, total corneal astigmatism; AA, anterior corneal astigmatism; PA, posterior corneal astigmatism; ACC, anterior corneal curvature; PCC, posterior cornea curvature; TCC, total corneal curvature.

## 4 Discussion

Senile cataracts are the main cause of visual impairment in elderly patients, and phacoemulsification combined with IOL implantation was the main clinical treatment for cataracts, effectively improving the patient’s vision. With the advancement of medicine and the increasing expectations of elderly patients for postoperative visual quality, more and more elderly patients choose multifocal IOL or Toric IOL in order to achieve better visual quality. Accurate measurement of corneal biological parameters was also a necessary condition to ensure the quality of cataract surgery, and as age increases, corneal biomechanics decreases (16). According to statistics, 41.8% of patients over 60 years elder in Hong Kong have astigmatism greater than 1.0 D, indicating that the likelihood of patients needing astigmatism correction increases with age (17). Therefore, it is particularly important to evaluate and understand the characteristics of curvature and astigmatism changes in elderly patients before and after cataract surgery, which has important guiding significance for the selection of IOL and the treatment of cataracts. In cataract patients, some clinical scholars currently believe that the influence of corneal posterior surface refractive power and posterior surface astigmatism on total corneal refractive power and total astigmatism is relatively small and can be ignored, while others believe that ignoring posterior surface curvature may lead to differences in clinically significant corneal curvature estimates (18, 19). In the past, corneal curvature and astigmatism values were mostly measured using corneal topography. The corneal topography measuring instrument designed based on the Placido disc uniformly projects 28 circular rings onto the corneal surface through a projection system, and converts them based on the mirror reflection angle of the anterior corneal surface. The corneal curvature values at the same location may vary due to different measurement directions and reference point axes, and the influence of corneal posterior surface curvature is ignored. In this study, the Sirius anterior segment analysis system was used, which combines the Placido ring with the Scheimpflug camera to accurately measure corneal thickness, total corneal refractive power, corneal anterior and posterior surface curvature radius, and high repeatability (11). Currently, with the increasing aging population, more and more patients require phacoemulsification surgery for cataracts, while corneal biomechanical properties were decreased with age. The postoperative corneal changes in elderly patients are not yet clear. Therefore, through this three-dimensional function, it is possible to effectively understand the preoperative and postoperative eye conditions of elderly patients, providing reference for surgical incision construction, intraoperative energy use, and preoperative IOL selection.

This study analyzed the changes in corneal curvature before and after surgery in elderly cataract patients. The total corneal curvature and anterior surface curvature increased compared to before surgery at 3 months after surgery. The curvature of the posterior surface of the cornea showed no significant difference compared to preoperative values. Spearman correlation analysis showed that the total curvature of the cornea was significantly positively correlated with the anterior surface curvature, and significantly negatively correlated with the posterior surface curvature. In addition, although previous studies have found that disregarding the corneal curvature of the posterior surface during preoperative IOL calculation and surgical design can have a certain impact on the postoperative corneal refractive state (20, 21). This study found that the curvature of the posterior surface of the cornea did not change significantly. This is consistent with previous research findings that cataract surgery has a relatively small impact on the posterior surface of the cornea (22). Some studies have also found that the influence of posterior corneal curvature in Barrett formula and Kane formula for measuring the power of artificial lenses was minimal (18). This study found that there was no significant difference in corneal anterior surface astigmatism, posterior surface astigmatism, and total astigmatism after 3 months of surgery compared to before. The reason for this was considered to be due to differences in surgical incision size and method. This study used a 2.2 mm transparent corneal incision. Previous studies found that the incidence of corneal endothelial dislocation, high-order aberration, and degree of corneal edema after surgery with a 2.2 mm corneal incision was lower, and the recovery was faster (23, 24). These results indicate that the surgical induced astigmatism caused by a transparent 2.2 mm corneal incision is relatively small. The Spearman correlation analysis results show that elderly cataract patients were prone to increased astigmatism after surgery with the growth of the axial length. The possible reasons for this may be: Long axial length elder patients have a decrease in corneal biomechanics, thinning of corneal thickness, and are prone to corneal deformation (25, 26); Long axial length elder patients have a decrease in corneal endothelial density and changes in cell morphology, which can lead to postoperative corneal edema and even decompensation (27, 28). Therefore, the repeated entry of the handle and the use of high energy should be minimized as much as possible to minimize the impact on the cornea during surgery.

Due to the small sample size of this study, there are certain limitations in the research results. Firstly, the changes in corneal curvature and astigmatism after surgery have not been dynamically observed. Secondly, the inclusion of patients with long axial length was small, which may lead to bias. Thirdly, the impact of postoperative corneal incision morphology and phacoemulsification time on corneal astigmatism has not been evaluated, and the simple surgical time may be biased. Fourthly, for standardization, all corneal incisions are performed at the 10 o’clock position. The results of this study may not reflect astigmatism induced by other corneal quadrants. In the future, a large sample size cross-sectional survey is needed for in-depth research, in order to further improve the postoperative visual quality of elderly patients with refractive cataracts and reduce refractive errors.

In summary, for elderly cataract patients with long axial length, the choice of multifocal intraocular lens should be carefully considered to avoid an increase in postoperative corneal astigmatism that may affect the surgical outcome. The Sirius anterior segment analysis system can accurately evaluate the dynamic changes in corneal curvature and astigmatism before and after surgery, providing a basis for the selection of artificial lenses, surgical incision construction, and intraoperative use of ultrasound energy, which has great clinical significance.

## Data Availability

The original contributions presented in the study are included in the article/supplementary material, further inquiries can be directed to the corresponding author/s.
